# Clinical effect of toothpaste and mouth rinse containing zinc lactate on oral malodor reduction

**DOI:** 10.4317/jced.55418

**Published:** 2019-04-01

**Authors:** Patcharawan Srisilapanan, Jeffrey Roseman, Tanrada Likitsatian

**Affiliations:** 1 Faculty of Dentistry, Nation University, Thailand; 2 Professor Emeritus, Department of Epidemiology, UAB School of Public Health, University of Alabama at Birmingham, Birmingham, Alabama, USA; 3 Postgraduate Student, Faculty of Medicine, Chiang Mai University, Thailand

## Abstract

**Background:**

This study aimed to investigate the effect of toothpaste and mouth rinse containing 0.14% zinc lactate on the reduction of three volatile oral malodor gases.

**Material and Methods:**

Ten subjects with good health were recruited to take part in a crossover design study with a 7-day washout period. They were randomly assigned to use the test (toothpaste and rinse containing 0.14% zinc lactate) or placebo (negative control) treatment regimens within the two-week period. All subjects were asked to refrain from tooth brushing and eating in the morning prior to the gas collection periods. The assessment of malodor measured the quantity of three volatile sulphur compounds (VSCs) using an OralChromaTM device. Oral gas collecting was divided into four time periods; before breakfast and the morning oral hygiene practice (baseline); after oral hygiene at 30 minutes, 1 hour and 2 hours. After the baseline assessment, each subject used the test or placebo treatment regimen for 7 days. After 7 and 14 days, subjects returned to the study site to repeat the same procedures with different products. Kruskal-Wallis was used to analyze the mean differences of malodor gases between the two test regimens.

**Results:**

The baseline mean of total VSCs in test and control groups was 6.5±3.7 and 1.7±9.3 ng/10 ml, respectively. The percent reduction of H2S at 30 minutes, 1 hour and 2 hours was statistically significant (*p*<0.005) in both treatments. The percent reduction of (CH3)2S and total VSCs in both treatments after 1 hour was statistically significant (*p*<0.005).

**Conclusions:**

The test treatment regimen was more effective than the placebo treatment regimen.

** Key words:**Zinc lactate, molodor, volatile sulphur compound, mouth rinse, toothpaste.

## Introduction

Oral malodor or halitosis is an important oral health problem affecting one-third of the world population (1). Intra oral cavities are the main cause of oral malodor (2,3). Due to decreased saliva secretion while sleeping, oral malodor after awakening is commonly reported (4). Microbial accumulation is the most important factor contributing to malodor production (5,6). Persistent malodor is primarily the result of microbial metabolism (6). Metabolism from gram-negative bacteria in the oral cavity produces gases with volatile sulphur compounds (VSCs) (7). Sulphur-containing amino acids transfer human proteins such as methionine, and cysteine into hydrogen sulphide (H2S), methyl mercaptan (CH3SH) and dimethyl sulphide ((CH3)2S) which are the three major components of VSCs (4,8-10). VSC production has been shown to be directly correlated with thiol and disulfide content in saliva (11). Oral bacteria from tongue coating, gingivitis and periodontitis can increase malodor (12-14). Oral malodor may cause social embarrassment. Several oral care products for reducing oral malodor have been developed including toothpaste and mouth rinse containing anti-bacterial properties such as cetylpyridinium chloride (CPC) and chlorhexidine. These products inhibit the mechanism of bacteria (14-17).

Certain metal ions such as zinc in the form of zinc salts have been added to oral hygiene products to reduce oral malodor, as well as control plaque and calculus (18). Zinc can inhibit the formation of VSC and reduce or inhibit oral malodor (19-21). Zinc ions, either as an aqueous solution or as dissolvable tablets interact with the sulfur in the substrate or in precursors of VSC to form insoluble sulfide. As a result, the malodor from sulphides is reduced (19). Moreover, zinc phosphate restrain the microbial activity by reacting with H2S gas (14,22-23). Sterer *et al.* tested palatal mucoadhesive tablets containing zinc in healthy adults and reported moderate significant reduction in malodor and VSC production (24). Zinc salt, therefore, has a good potential in reducing malodor when added into oral hygiene products.

This study aimed to investigate the effect of toothpaste and mouth rinse containing zinc salt or 0.4% zinc lactate on the reduction of volatile oral malodor gases compared to placebo toothpaste and mouth rinse.

## Material and Methods

-Study design and subjects

This study was a randomized, double-blinded, crossover clinical trial. Ten volunteer subjects aged between 20 to 50 years old were recruited. They were screened and found to have higher levels of three volatile sulphur compounds than the cut-points. Cut-points were (H2S)>1.5ng/10mL for hydrogen sulphide, (CH3SH)>0.5ng/10mL for methyl mercaptan and ((CH3)2S)>0.2ng/10mL for dimethyl sulphide. Subjects did not have medical history of infectious diseases (e.g. hepatitis, HIV and tuberculosis), and had no periodontal diseases. The subjects had not undergone a course of antibiotics and/or anti-inflammatory drugs one month prior to the start of the trial or during the trial.

Ten malodor subjects were randomly assigned to use the test or placebo treatment regimens within the two-week period. The test treatment regimens consisted of tooth brushing and mouth rinsing with the product containing 0.14% zinc lactate. The placebo treatment regimens consisted of tooth brushing and mouth rinsing with toothpaste containing no zinc lactate and mouth rinse containing no zinc lactate.

Randomization for the crossover design was done by the statistician using the manual randomization sequence. The statistician did not have a role in the treatment of the subjects All mouthwash bottles were identical with plain label. Each subject was randomly given a bottle of the test or placebo mouthwash by an operator who was blinded to the type of the mouthwash in each bottle.

-Washout periods

Before the study periods, the subjects were asked to brush their teeth with Fluocaril® original (placebo) toothpaste twice a day for 7 days.

-Oral gas collecting and analysis

On the gas collection day, subjects were asked to refrain from tooth brushing and eating in the morning prior to the gas collection periods. Moreover, they were not allowed to change their oral hygiene practices during the trial, and restrained from consuming foods associated with oral malodor (such as garlic, onions, spicy foods and alcohol beverages) beginning one day prior to the start of the trial and continuing for the duration of the trial. On the sampling day, the volunteers were not allowed to use strong smelling perfumes, cosmetics, lipstick or hairspray. Dentures had to be removed during gas collection.

**Figure 1:**
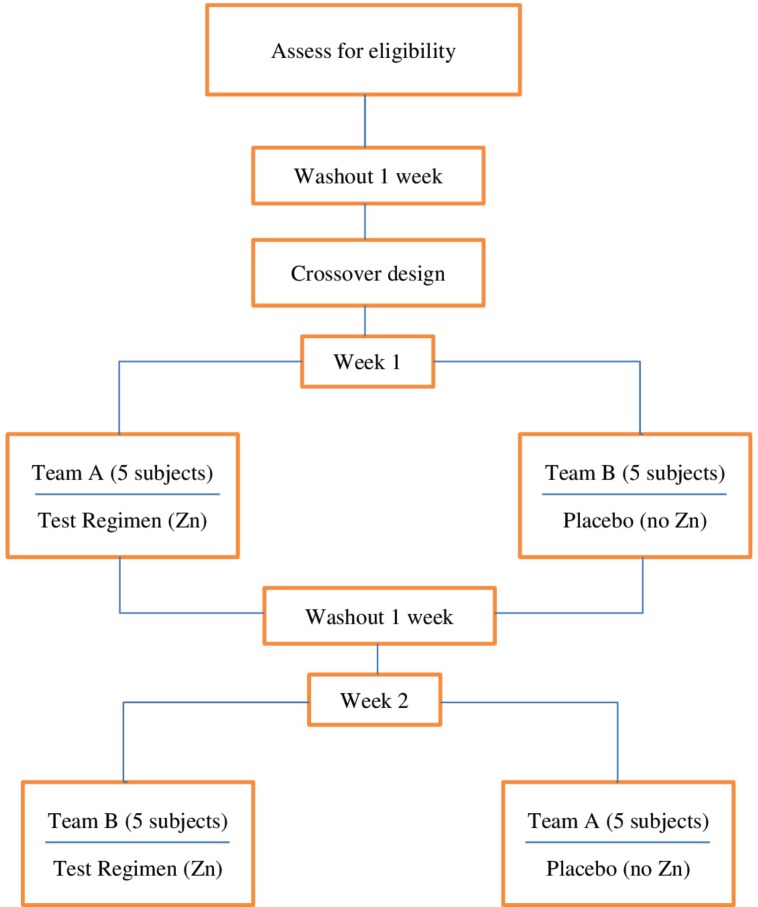
The flow diagram of the study.

At the beginning of the first day of the study periods, the baseline oral gases were collected at four time periods. The first collecting at the study site was the gas before the morning oral hygiene practice and before having breakfast. After the first gas collection, each subject used the assigned products. The second gas collection was collected 30 minutes after the baseline collection. Subjects were asked to remain at the study site during the interval between each gas collection period. The third and fourth gas collection was done 60 and 120 minutes after the baseline. After that, each subject used the test or placebo treatment regimen for 7 days. After 7 days, subjects returned to the study site to repeat the same procedures but changed the products. Those who used the test treatment regimens changed to the placebo treatment regimen and vice versa. Figure 1 illustrates the flow diagram of the study.

OralChromaTM was used to measure the quantity of the three volatile sulphur compounds (VSCs), which are the major compounds contributing oral malodor. The major three VSCs are hydrogen sulfide (H2S), methyl mercaptan (CH3SH) and dimethylsulfide ((CH3)2S).

According to Tangerman and Winkel (25), Oral Chroma has been tested and accepted as the method of choice to detect malodorous gases. It has been widely used in other studies (26,27).

The oral gas was collected using a 1 ml syringe. The syringe was inserted into the oral cavity until the flange reached the lips. The subject was asked to softly bite on the syringe and close the mouth tightly for 30 seconds. Then, the piston was pulled to the very end of the syringe. The breath sample was filled in to the syringe and immediately ejected out by pushing the piston to the flange of syringe 2 times to eliminate unwanted air in the syringe. Finally, the oral gas was collected by pulling the piston until the plunger reached 1 mL and then directly injected into OralChromaTM. The quantities of the three VSCs were displayed after a 4-minute gas analysis.

-Statistical Analysis

A Kruskal-Wallis test was used to analyze the differences in the amount of VSCs at each period between test and placebo treatment regimens.

-Ethical Consideration

This study was approved by the Ethical Committee of the Faculty of Dentistry, Chiang Mai University.

## Results

Subjects consisted of 5 males and 5 females. The mean age was 30.7 ±11.6 years.

At 30 minutes after using the treatment regimens, the percent reduction of mean H2S in the group using toothpaste and rinsing without zinc lactate (placebo treatment) was 25.7%, and 98.3% for the group using toothpaste and rinsing with zinc lactate (test treatment). The percent reduction of mean CH3SH was 53.2% for the placebo group and 71.3% for the treatment group, and for (CH3)2S it was -18.5% for the treatment group and -31.8% for the placebo group. The total percent reduction of VSCs for all treatments was 21.2% for the placebo group and 50.2% for the treatment group (Fig. 1).

At 1 hour after using the treatment regimens, the percent reduction of mean H2S was 7.8% for the placebo group and 85.4% for the treatment group. For CH3SH it was 36.1% for the placebo group and 58.5% for the treatment group. For (CH3)2S it was -22.4% for the placebo group and 65.3% for the treatment group. The total percent reduction of VSC from all treatments was 8.0% for the placebo group and 70.9% for the treatment group (Fig. 1).

At 2 hours after using the treatment regimens, a mean percent reduction was observed in all three volatile gases and total VSC (Fig. 1).

**Figure 2:**
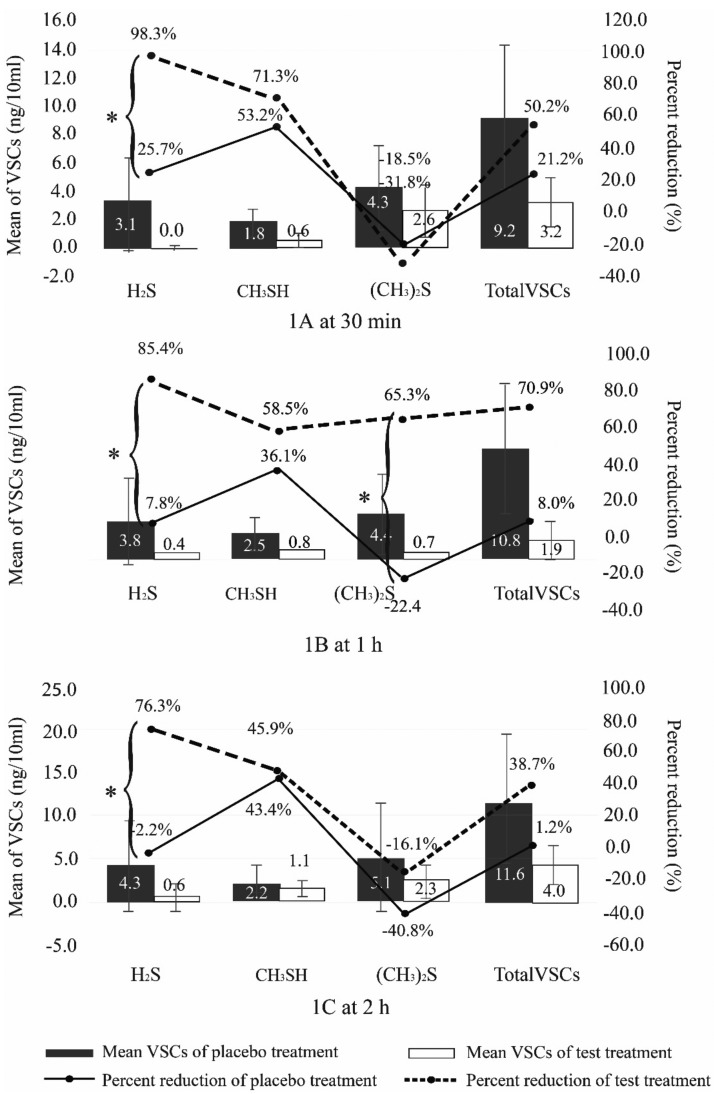
Comparison of mean and percent reduction of three malodor gasses and total VSCs between test and placebo treatment regimens at three time periods after oral hygiene.

A Kruskal-Wallis test showed that at baseline, there was no significant different between the mean of test and control of all three gases and total VSCs ( Table 1). However, the percentage reduction of VSC in both treatment groups was statistically significant for H2S at all time-points (30 min; *p*=<0.001, 1 h; *p*=0.001 and 2 h; *p*=0.002). There was statistically significant reduction of (CH3)2S (*p*=0.002) and total VSCs (*p*=0.000) at 1 h ( Table 1, Fig. 2).

**Table 1:**
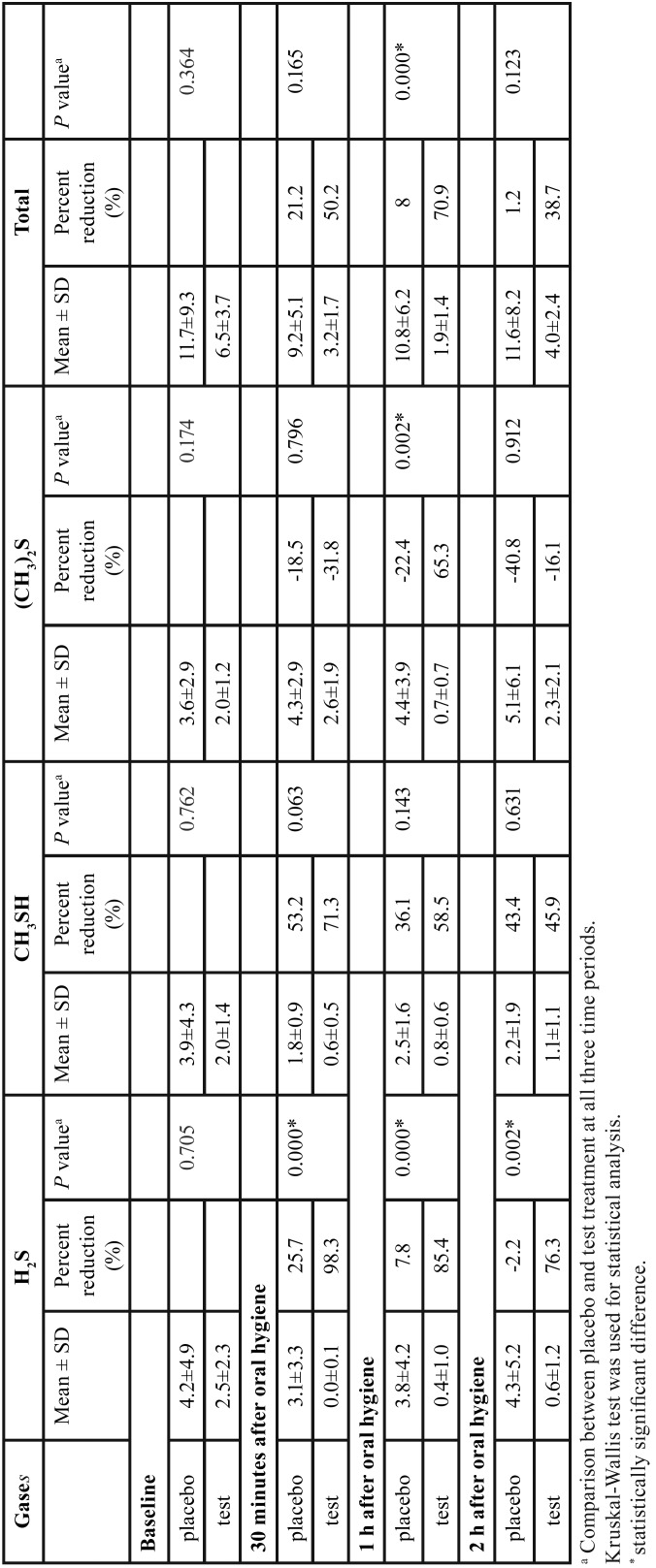
Means of the three volatile gases and total VSCs at baseline and at three time periods.

## Discussion

In this randomized, double-blinded, crossover clinical trial, treatment regimens containing 0.14 % zinc lactate showed a higher reduction of the three volatiles gases and total VSCs than the placebo treatment regimen. The reduction of H2S at the three time periods was significantly different compared to the placebo, while the reduction of mean (CH3)2S and total VSCs were statistically significant compared to the placebo at 1 hour.

This present study confirms that zinc salts can reduce malodor when added into oral hygiene products such as toothpaste and mouthwash. There have been a number of other studies which shown the effect of zinc salts in mouthwash to reduce volatile gases in oral cavity (18,24,25,27). Young *et al.* (18) reported 1% zinc acetate solution had excellent anti-VSC effect throughout the test period of 3 hours. Sterer *et al.* reported zinc salt added into the palatal muco-adhesive tablet reduced malodor and VSC production (24). Zinc chloride plus sodium chlorite mouthrinse was shown to be more effective in reducing oral malodor than the mouthrinse containing no zinc chloride/no sodium chlorite (25). A meta analysis of randomized control trials to test the effect of zinc salt on reducing malodor gases indicated that zinc containing mouthrinses can be effective in neutralisation of odouriferous sulphur compounds (26). A recent study by Mendes *et al.* (27) using two different mouth rinses, one containing 0.14% zinc lactate and the other containing 0.18%, zinc pidolate, among other factors, found both mouth rinses reduced VSC at 1, 3, and 5 hour test intervals.

This study had a number of limitations. The sample size was small. Despite this many of the differences were significant. The measurement of volatile sulphur compounds (VSCs) is sensitive to associated psychosocial factors such as subject health status, stress and economical status. These unmeasured factors could contribute to the variability of VSCs levels within the same subject (28). According to Tangerman and Winkel (28) Oral Chroma has been tested and accepted as the method of choice to detect malodor gases. It has been widely used in other studies (29). However, there may be some false outcome measures in the use of the OralChromaTM system. The concentrations given by the OralChromaTM for the different VSCs could be incorrect if there is an incorrect assignment of the position of the VSCs in the chromatogram (28).

Despite these limitations, this and similar studies suggest zinc salts in oral hygiene products such as toothpaste and mouth rinse show good potential for reducing malodor.

## Conclusions

The combination of 0.14% zinc lactate in toothpaste and mouth rinse reduced the three volatile gases and total VSC at all assessment times. This result and those of similar studies suggest zinc salts in oral hygiene products such as toothpaste and mouth rinse show potential for reducing malodor.
